# The prognostic value of Clinical Frailty Scale in COVID-19 pneumonia across different pandemic phases: a comparison between the first and the fourth wave

**DOI:** 10.3389/fmed.2025.1549444

**Published:** 2025-03-27

**Authors:** Carmine Siniscalchi, Andrea Ticinesi, Angela Guerra, Alberto Parise, Nicoletta Cerundolo, Beatrice Prati, Riccardo Simoni, Emanuela Porro, Tiziana Meschi

**Affiliations:** ^1^Department of Medicine and Surgery, University of Parma, Parma, Italy; ^2^Geriatric-Rehabilitation Department, Azienda Ospedaliero-Universitaria di Parma, Parma, Italy

**Keywords:** frailty, COVID-19, vaccine, death, pandemic COVID-19, prognostic

## Abstract

**Introduction:**

The COVID-19 pandemic has placed unprecedented strain on health-care systems. Frailty is being used in clinical decision making for patients with COVID-19, yet the prevalence and effect of frailty in people with COVID-19 may be influenced by the local characteristics of each pandemic wave. We aimed to establish the prevalence of frailty in older patients with COVID-19 who were admitted to hospital and investigate its association with mortality comparing non-vaccinated patients of the first wave versus vaccinated patients in the fourth wave.

**Materials and methods:**

This was an observational study conducted at one single hospital center in Italy. All older adults (≥70 years) admitted with confirmed COVID-19 (positive molecular testing) were included. Data of 658 patients (493 non-vaccinated COVID-19 patients admitted during the first wave and 165 patients vaccinated against COVID-19 during the fourth wave), were collected from clinical records including symptom type, extension of lung abnormalities on chest computed tomography (CT), laboratory parameters. Frailty was assessed by Clinical Frailty Scale (CFS) and patients were grouped according to their score (≤4: fit or pre-frail; 5–6 = initial signs of frailty but with some degree of independence; >7 = severe or very severe frailty). The primary outcome was in-hospital mortality.

**Results:**

In comparison with vaccinated patients from the fourth wave, unvaccinated patients from the first wave had reduced prevalence of heart disease (35% vs. 56%), renal failure (9% vs. 15%), but higher prevalence of fever at time of diagnosis (84% vs. 59%), malignancy (16% vs. 6%), higher computed tomography (CT) severity visual score, higher CRP (C-reactive protein) serum levels (median value 105 mg/L vs. 75 mg/L), but lower burden of frailty. In a stepwise multivariable logistic regression model, unvaccinated patients from the first wave had a higher risk of death regardless of CFS [Odds Ratio (OR) 2.241, 95% confidence interval (CI) 1.492–3.336, *p* < = 0.001], while in the fourth wave, CFS was significantly associated with hospital mortality.

**Conclusion:**

Our study suggests that in non-vaccinated older patients from the first pandemic wave CFS was unable to stratify the risk of death.

## Introduction

SARS-CoV-2 is a viral respiratory tract infection caused by SARS-CoV-2 that led to a pandemic in early 2020 in Western countries after spreading from China. Several studies, clinical trial and case series are being published for describe the clinical features and predictors of mortality in patients with COVID-19 (1, 2). In these studies, older age has consistently been shown to be associated with poor outcomes and increasing mortality (3). Frailty is a geriatric syndrome associated with poor prognosis in all acute and chronic illnesses. Recently the National Institute for Health and Care Excellence suggested the use of frailty indicators, such as the Clinical Frailty Scale (CFS), also for hospitalized COVID-19 patients (4, 5). However, the National Health Service Specialist Clinical Frailty Network recommended that CFS should not be used alone for critical hospitalized patients, but the prognostic stratification must be taken in conjunction with disease-specific scores, and that the guidance might not apply to younger people or those with particular illness and disabilities. An important research gap with regards to supporting the use of CFS in the acute management of SARS-COV2 patients still remains.

Frailty is defined as “a medical syndrome with multiple causes and contributors that is characterized by diminished strength, endurance, and reduced physiologic function that increases an individual’s vulnerability for developing increased dependency and/or death” (6). The prevalence of frailty in middle-aged and older patients varies according to the method of assessment and the specific population but is estimated to be about 40% (7, 8). The likelihood of being frail increases with age, but can occur in younger adults (9). In addition, there is substantial evidence that frailty equates to worse patient outcomes in those admitted to hospital, including medical and surgical admissions as well as patients requiring intensive care (10). Data on the prevalence of frailty in hospitalized patients with COVID-19 and its prognostic value are discordant, depending on the setting and organization of care during the different pandemic waves (11). The aim of our study was to establish whether the CFS was able to stratify the risk of death in hospitalized patients for COVID-19 and if are any differences in this relationship across different waves (first wave with unvaccinated patients versus fourth wave with predominantly vaccinated patients).

## Materials and methods

### Patient characteristics and data collection

This study was conducted in an Internal Medicine Unit of a large teaching hospital in Northern Italy (Parma University-Hospital), that has been appointed as the main hub for the care of SARS-CoV-2 patients of the whole Parma province (approximately 450,000 inhabitants) since the earliest phases of the first wave (12, 13). Two groups of patients hospitalized with SARS-CoV-2 were retrospectively enrolled after check for inclusion and exclusion criteria and availability of data on clinical records. The two groups corresponding to the first pandemic wave from Mars to May 2020 and the fourth pandemic wave from October to December 2021, including, respectively, 493 patients and 165 patients. This subclassifications were made to distinguish the first period characterized by 100% of patients without a specific vaccine for COVID-19, from the fourth wave in which all patients had received a vaccine against COVID-19 at the moment of admission.

Only patients aged ≥70 years old with SARS-CoV-2 infection confirmed by reverse transcriptase polymerase-chain reaction (RT-PCR) on nasopharyngeal swab performed upon urgent admission were included in the study. Conversely, subjects with missing data on virological and radiological variables and subjects who were transferred to other wards (i.e., with missing data on outcome) were excluded from the study.

The records of each participant were reviewed in order to collect demographic data (age and sex), number and types of comorbidities (including hypertension, diabetes, obesity, dyslipidemia, heart diseases, cancer, chronic kidney disease, dementia), clinical presentation of SARS-CoV-2 (vital signs, chest CT abnormalities) and the results of lab tests performed on admission, including arterial blood gas analysis, blood cell count, D- dimer, CRP and procalcitonin (PCT). The extension of pulmonary infiltrates and abnormalities on chest CT was estimated through calculation of the chest CT visual score, detailed elsewhere (14). We also evaluate the frailty for all enrolled patients using a global clinical measure of fitness and frailty in elderly people, the Clinical Frailty Scale (CFS) developed by Kenneth Rockwood and colleagues (5). This tool is widely used in clinical practice and research for the evaluation of frailty according to the deficit accumulation model, and validated in the scientific literature for multiple clinical settings, ranging from critical care to primary care outpatients (15, 16).

Data on outcome (survival vs. death) were also collected for all participants.

Ethics Committee approval was obtained (Comitato Etico dell’Area Vasta Emilia Nord, Emilia-Romagna region) under the IDs 273/2020/OSS/AOUPR and 959/2021/OSS/AOUPR as part of a larger projects on the characteristics of patients hospitalized with confirmed or suspect COVID-19. All participants, who were contactable by phone or for follow-up reasons, provided written informed consent for participations. For all other cases, the Ethics Committee, in accordance with the guidelines in force at the moment of approval, waived written informed consent collection due to retrospective design of the study.

### Statistical analyses

Variables were expressed as median and interquartile range (IQR) or percentages, as appropriate. The characteristics of participants were compared with the Mann–Whitney or Kruskal-Wallis or chi-square tests. *P* for trend calculated with Jonckheere Terpstra or Mantel Haenszel tests. Kaplan–Meier analysis and Cox regression were used for survival curves. The factors independently associated with death in both groups were investigated with stepwise multivariate logistic regression models considering participants altogether and after partition by pandemic wave and use of vaccine. Additional analyses were also made after categorization of participants according to pandemic wave and vaccination status. Analyses were performed with the SPSS statistical package (v. 29, IMB, Armonk, US), considering *p* values <0.05 as statistically significant.

## Results

We included in this study 658 patients, 493 non-vaccinated patients (first pandemic wave period from March to May 2020) and 165 vaccinated patients (fourth pandemic wave from October to December 2021). Their clinical characteristics are compared in Table 1. Non- vaccinated patients had less comorbidities than the vaccinated ones. The unvaccinated patients from the first wave had lower incidence of obesity than the vaccinated ones from the fourth wave. However, obesity represented a risk factor for mortality in unvaccinated patients rather than the vaccinated ones. The unvaccinated patients had higher pulmonary impairment evaluated through Computed Tomography “visual score” for COVID19 pneumonia (median 30% vs. 20%), they had worse calculated fractional inspired oxygen saturation on admission (PaO2/FiO2) (median value 205 mmHg vs. 281 mmHg), higher C-reactive protein (CRP) (median value 105 mg/L vs. 75 mg/L), higher Procalcitonin serum levels (PCT) (median value 0.21 ng/mL vs. 0.14 ng/mL), but were suffering less often from chronic heart disease (prevalence 35% vs. 56%), renal failure (prevalence 9% vs. 15%) and chronic diseases in general (median 3 vs. 5). When stratifying for three groups of CFS intervals (≤ 4 points, 5–6 points, ≥7 points respectively), unvaccinated patients presented similar PCR value (*p* = 0.193), PCT (*p* = 0.600), PaO2/FiO2 (*p* = 0.799) between groups and similar mortality rate (*p* = 0.463) (Table 2, a). Vaccinated patients with CFS ≤ 4 had higher PaO2/FiO2 (*p* = 0.004) and a lower PCT in comparison to vaccinated patients with CSF >7 points (Table 2, b). Unvaccinated patients with CFS ≤4 had higher risk to die in comparison with vaccinated patients (*p* < 0.001), while for patients with CFS score of 5–6 (*p* = 0.084) or for patients with CFS score of ≥7 (*p* = 0.409) no statistical differences were found (Table 3). Using a Cox regression model testing the CFS parameter associated with in hospital mortality, only for vaccinated patients CFS was able to identify patients at risk to die (Table 4). Lactate dehydrogenase (LDH) in patients group with CFS ≤4 was 340 as medium value (with range from 250 to 430), in patients group with CFS 5–6 was 333 (261–459) and in patients group with CFS ≥ 7 was 320 (241–428), with no statistically significant trends across CFS categories.

**Table 1:** Comparison of demographic, anamnestic, and clinical characteristics of patients with COVID-19 pneumonia stratified by pandemic wave.

N. 658	1 wave (March–May 2020) N.493	4 wave (October–December 2021) N.165	*p*
Age, years	80 (75–86)	82 (77–87)	0.067
Patients vaccinated for COVID-19, %	0	100	/
Female gender, %	46	45	0.866
CSF	5 (3–6)	5 (4–7)	**0.024**
Chronic diseases, *n*	3 (2–5)	5 (3–7)	**<0.001**
Chronic heart disease, %	35	56	**<0.001**
Hypertension, %	71	66	0.302
Obesity, %	7	9	0.346
Diabetes, %	24	20	0.261
Dyslipidemia, %	21	20	0.780
IRC, %	9	15	**0.041**
Dementia, %	21	28	0.074
Neoplasia, %	16	6	**0.004**
Fever, %	84	59	**<0.001**
CT visual score, %	30 (20–50)	20 (10–35)	**<0.001**
PaO_2_/FiO_2_, mmHg	205 (108–309)	281 (242–331)	**<0.001**
Hemoglobin, g/dL	13.5 (12.0–14.6)	12.8 (11.3–13.9)	**<0.001**
D-Dimer, ng/mL	1,137 (750–2,147)	903 (581–1,552)	**<0.001**
Lymphocytes, mm^3^	808 (559–1,149)	896 (614–1,364)	**0.021**
C-reactive protein, mg/L	105 (56–173)	75 (33–131)	**<0.001**
Procalcitonin, ng/mL	0.21 (0.09–0.55)	0.14 (0.07–0.44)	**0.021**
Length of stay	6 (3–11)	17 (9–29)	**<0.001**
Death, %	44	31	**0.003**

Data expressed as median and IQR or percentage. *p* values calculated with Mann–Whitney for continuous variables and chi-square test for dichotomous variables. *p* < 0.05 are indicated in bold.

**Table 2:** Comparison of demographic, anamnestic and clinical characteristics of patients with COVID-19 pneumonia stratified by unvaccinated-first wave (a) and vaccinated -fourth wave (b) and CSF ≤ 4, 5–6 e ≥ 7.

(a) Unvaccinated-first wave patients (N.493)					
	CSF ≤4 N.220	CSF 5–6 N.156	CSF ≥7 N.117	*p*	*p* for trend
Age, years	77 (73–81)	82 (78–87)	86 (82–89)	**<0.001**	**<0.001**
Patients admitted per day, number	26 (19–30)	20 (15–30)	17 (6–27)	**<0.001**	**<0.001**
Female gender, %	42	38	65	**<0.001**	**<0.001**
Chronic diseases, *n*	3 (2–4)	4 (3–5)	4 (3–6)	**<0.001**	**<0.001**
Chronic heart disease, %	27	39	44	**0.004**	**0.001**
Hypertension, %	67	77	70	0.082	0.317
Obesity, %	8	8	3	0.105	0.080
Diabetes, %	21	26	25	0.555	0.402
Dyslipidemia, %	21	26	12	**0.013**	0.135
IRC, %	6	10	12	0.177	0.070
Dementia, %	1	19	62	**<0.001**	**<0.001**
Neoplasia, %	15	20	15	0.352	0.654
Fever, %	89	86	73	**<0.001**	**<0.001**
CT visual score, %	35 (25–55)	30 (15–50)	30 (15–50)	**0.019**	**0.011**
PaO_2_/FiO_2_, mmHg	200 (107–310)	223 (115–299)	197 (104–312)	0.799	0.722
Hemoglobin, g/dL	13.6 (12.4–14.7)	13.3 (11.8–14.5)	13.4 (11.5–14.6)	0.244	0.114
D-Dimer, ng/mL	1,099 (722–1860)	1,166 (754–2,284)	1,252 (828–2,347)	0.265	0.108
Lymphocytes, mm^3^	847 (565–1,137)	794 (569–1,195)	786 (524–1,164)	0.895	0.746
C-reactive protein, mg/L	105 (63–179)	114 (56–173)	91 (51–164)	0.193	0.166
Procalcitonin, ng/mL	0.19 (0.09–0.58)	0.23 (0.10–0.54)	0.21 (0.10–0.56)	0.600	0.333
Length of stay	7 (3–11)	6 (3–11)	6 (3–13)	0.825	0.986
Death, %	42	43	49	0.463	0.252

(b) Vaccinated-fourth wave patients (N.165)					
	CSF ≤4 N.48	CSF 5–6 N.77	CSF ≥7 N.40	*p*	*p* for trend
Age, years	76 (73–80)	83 (79–87)	86 (79–91)	**<0.001**	**<0.001**
Patients admitted per day, number	2 (1–4)	2 (2–4)	2 (1–3)	0.877	0.625
Female gender, %	37	53	40	0.146	0.659
Chronic diseases, *n*	4 (2–5)	5 (4–7)	6 (4–8)	**<0.001**	**<0.001**
Chronic heart disease, %	41	67	53	**0.013**	0.192
Hypertension, %	59	68	73	0.381	0.179
Obesity, %	17	5	8	0.093	0.114
Diabetes, %	24	17	18	0.565	0.386
Dyslipidemia, %	21	25	7	0.071	0.114
IRC, %	0	18	25	**0.001**	**<0.001**
Dementia, %	2	22	70	**<0.001**	**<0.001**
Neoplasia, %	0	10	4	0.137	0.347
Fever, %	67	60	48	0.166	0.062
CT visual score, %	20 (10–30)	20 (10–30)	25 (11–40)	0.407	0.200
PaO_2_/FiO_2_, mmHg	307 (265–363)	276 (250–315)	257 (190–300)	**0.004**	**<0.001**
Hemoglobin, g/dL	13.3 (12.3–14.3)	12.5 (11.1–13.9)	11.9 (10.8–13.7)	**0.020**	**0.005**
D-Dimer, ng/mL	603 (468–1,010)	1,062 (699–1,630)	1,162 (580–1828)	**0.004**	**0.002**
Lymphocytes, mm^3^	1,069 (700–1,425)	866 (578–1,320)	854 (510–1,255)	0.353	0.191
C-reactive protein, mg/L	67 (24–127)	65 (34–128)	91 (39–182)	0.144	0.086
Procalcitonin, ng/mL	0.10 (0.04–0.29)	0.13 (0.07–0.36)	0.40 (0.10–3.66)	**<0.001**	**<0.001**
Length of stay	14 (8–24)	19 (10–29)	22 (9–32)	0.111	0.052
Death, %	9	31	56	**<0.001**	**<0.001**

Data expressed as median and IQR or percentage. *p* values calculated with Kruskal-Wallis for continuous variables and chi-square test for dichotomous variables. *p* for trend calculated with Jonckheere Terpstra or Mantel Haenszel. *p* < 0.05 are indicated in bold.

**Table 3:** Death in patients from the 1st and 4th waves.

	1 wave	4 wave	*p*
Death in CSF patients ≤4, %	42	9	**<0.001**
Death in CSF 5–6 patients, %	43	31	0.084
Death in CSF patients ≥7, %	49	56	0.409

A highly significant difference is found between patients with Rockwood ≤4 in the 2 waves, while the difference with CSF 5–6 is at the limits of significance. Data expressed as percentage. *p* values calculated with chi-square test. *p* < 0.05 are indicated in bold.

**Table 4:** Cox regression model testing the CSF parameter associated with hospital mortality in the two pandemic waves.

Death	Significance	Odds ratio	95% CI for odds ratio
First wave			
CSF	0.892		
Fourth wave			
CSF	**0.010**		
CSF 5–6 vs. CSF ≤4	0.078	2.604	0.899–7.544
CSF ≥ 7 vs. CSF ≤4	**0.005**	4.586	1.574–13.362

*p < 0.05 are indicated in bold.*

On a stepwise multivariate logistic regression model (Table 5), age > 75 years (OR 2.075, 95% confidence interval, CI 1.313–3.279, *p* = 0.002) and male gender were (OR 1.698, 95% confidence interval, CI 1.175–2.454, *p* = 0.005), were independently associated with mortality during hospitalization in non-vaccinated patients. On a stepwise multivariate logistic regression model (Table 5), age (OR 1.070, 95% confidence interval, CI 1.008–1.135, *p* = 0.026), high number of chronic diseases (OR 1.254, 95% confidence interval, CI 1.061–1.481, *p* = 0.008) and CFS score (OR 1.552, 95% confidence interval, CI 1.118–2.156, *p* = 0.009) were independently associated with mortality during hospitalization in vaccinated patients.

**Table 5:** Factors associated with hospital mortality on stepwise multivariate logistic regression analysis, in the population with COVID-19 pneumonia divided by wave (covariates: age, sex, chronic diseases number and CSF).

Death	Significance	Odds Ratio	95% CI per Odds ratio
First wave			
Age > 75 years vs. Age ≤ 75 years	**0.002**	2.075	1.313–3.279
Males vs. Females	**0.005**	1.698	1.175–2.454
Fourth wave			
Age	**0.026**	1.070	1.008–1,135
Chronic diseases, number	**0.008**	1.254	1.061–1.481
CSF	**0.009**	1.552	1.118–2.156

*p < 0.05 are indicated in bold.*

**Figure 1:**
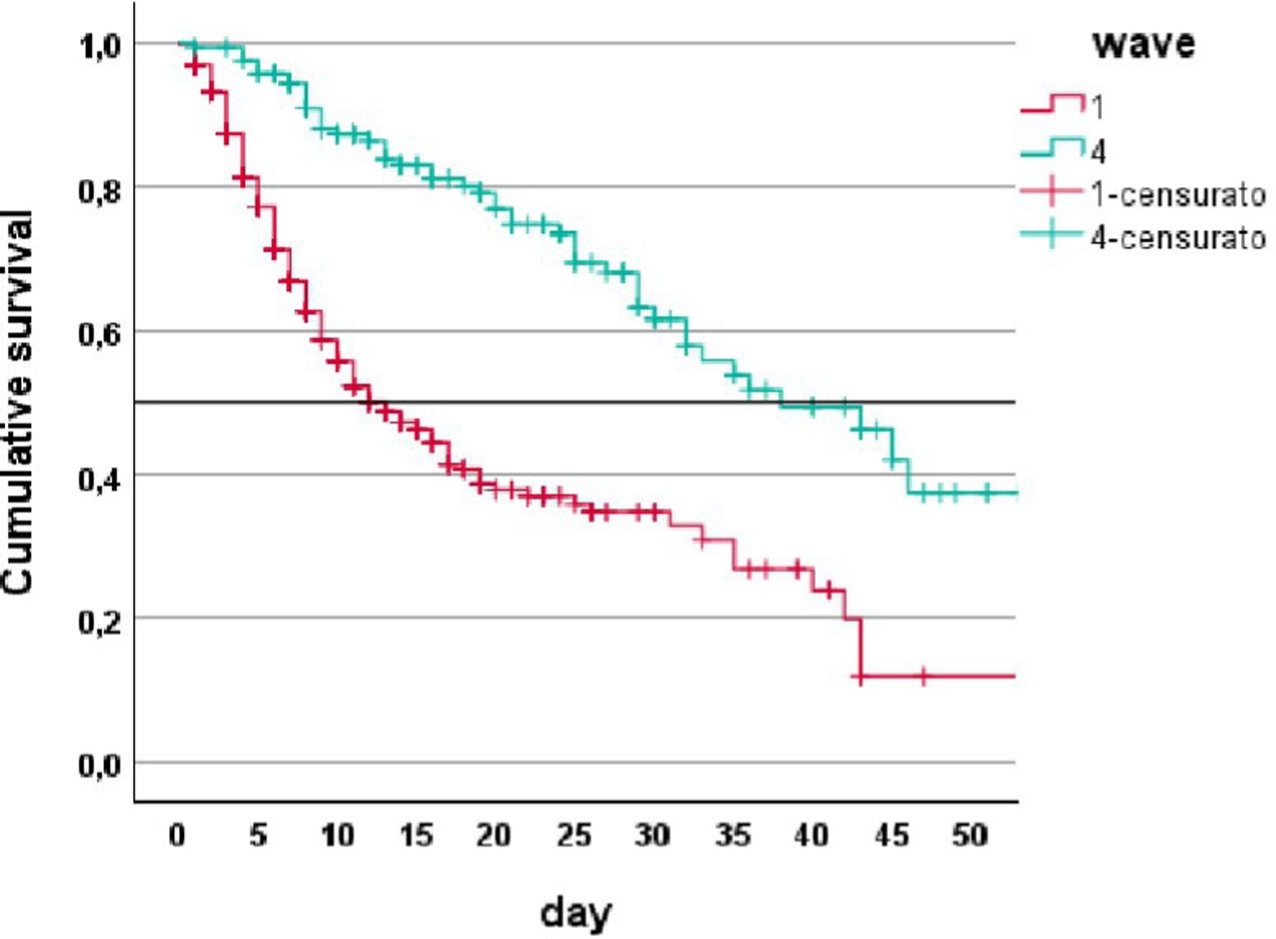
Cumulative survival (Kaplan–Meier analysis) in patients from first wave (1) and fourth wave (4).

Cumulative survival Kaplan–Meier analysis shows that vaccinated patients in the fourth wave had a 50% survival rate of 38 days of hospitalization vs. 13 days for unvaccinated patients, *p* < 0.001 (Figure 1).

**Figure 2:**
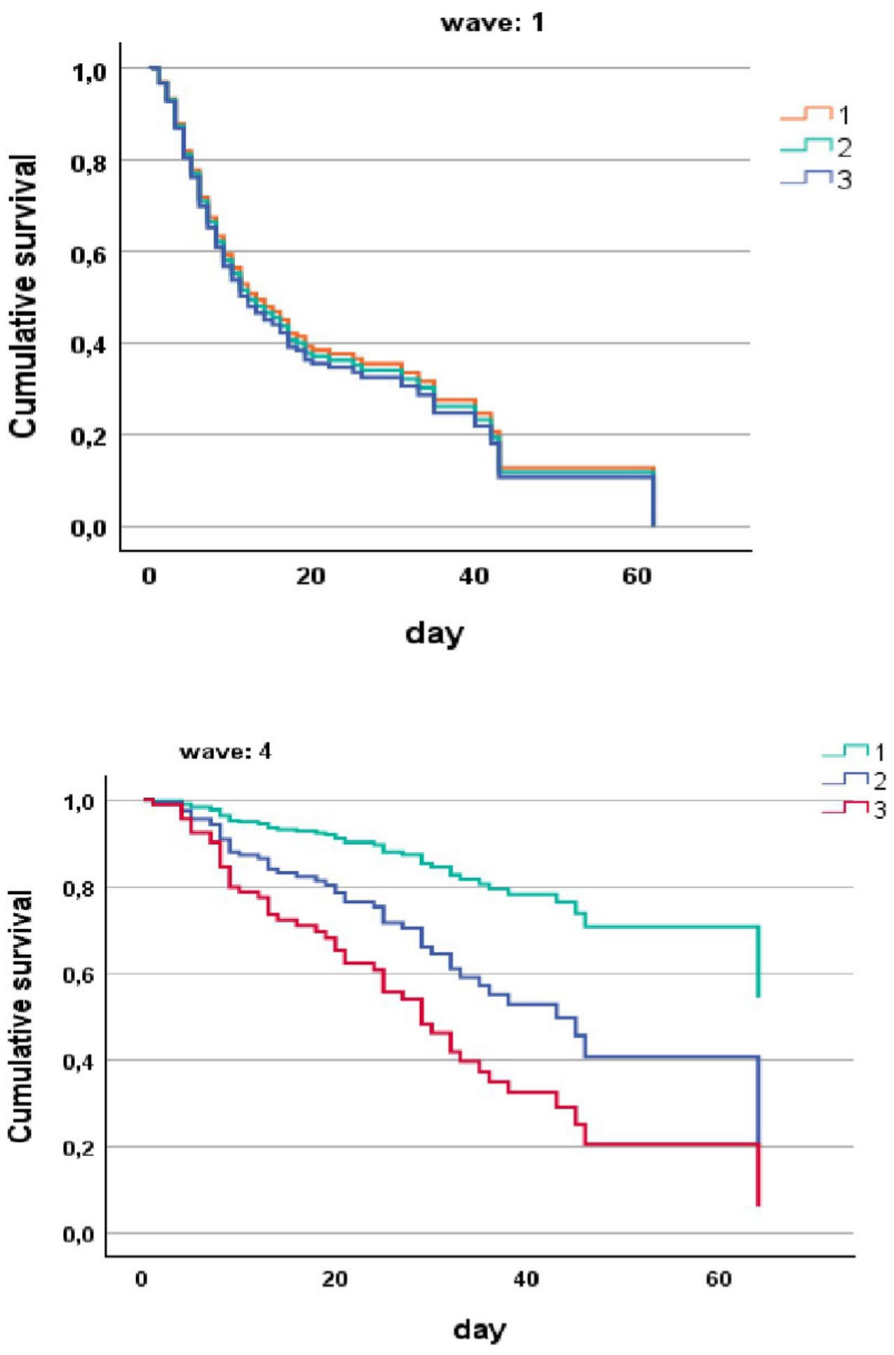
Risk of death in hospital (COX regression) in COVID-19 patients stratified by wave and by CFS ≤ 4 (1), CFS 5–6 (2), and CFS ≥ 7 (3).

In a Cox regression model for the risk of death in hospital in COVID-19 patients stratified by vaccine and by CFS score with cutoff of ≤4, 5–6 and ≥ 7, clearly shows that CFS was unable to stratify the risk of death in unvaccinated patients during the first pandemic waive (Figure 2). CFS scale was able to stratify the risk of death in vaccinated patients during the fourth wave (Figure 2).

## Discussion

In this retrospective study, we showed that, in our clinical and organizational setting, the CFS score was able to stratify the risk of death in vaccinated patients hospitalized for SARS-COV2 during the fourth pandemic wave, but not in unvaccinated older subjects admitted during the first pandemic wave. Despite the latter had a lower burden of comorbidities, the characteristics of COVID-19 infection appeared more severe, with worse CT visual score, higher laboratory inflammations marker (such as PCR and PCT) and worse PaO2/FiO2. The high capability of the CFS to predict the in hospital mortality has been already demonstrated in multicenter studies since the earliest phases of the first pandemic wave (17–27). For example, in a group of older patients with a median age of 85 years old admitted to a Geriatric Department of a general hospital in Belgium, CFS score, serum lactate dehydrogenase levels and viral load on nasopharyngeal swabs were the only significant predictors of mortality (18). In a large multicenter study conducted in the United Kingdom and Italy, CFS score in patients with confirmed COVID-19 infection of different age ranges was able to predict hospital mortality more accurately than age and comorbidities alone (17). Hägg et al. suggested that the addition of CFS evaluation to demographic characteristics and number of comorbidities was extremely accurate in stratifying prognostic risk in older patients during the first pandemic wave (19). Despite the overlap between multimorbidity and frailty, CFS score and number of chronic conditions remained both independently associated with hospital mortality in an Italian acute geriatric ward (20). The prognostic value of CFS, however, seemed to be reduced in adult patients younger than 65, according to an international multicenter study conducted across different European countries (21). The prognostic performance of CFS seemed even superior to that of the traditional 70-item frailty index, with the significant advantage of the very short time of completion (22), and allowed to identify a subgroup of geriatric patients with more severe pulmonary involvement (23). In a multicenter study from the Netherlands, the relationship between CFS and hospital mortality was mitigated by the circumstance that older frail patients tended to be admitted to the hospital at an earlier stage of the illness, with significantly less severe respiratory symptoms than younger individuals (24).

Some studies warned from the use of CFS as the only prediction tool for mortality risk in geriatric patients, highlighting the role of male gender (25), presence of fever and pulmonary involvement on chest radiograms (26). CFS was however able to predict not only in-hospital, but also post-discharge mortality in a large group of patients admitted with COVID-19 in an Italian hospital during the first and the second pandemic wave, before the availability of vaccines (27). Therefore, the state-of-the-art of the literature from the first pandemic wave indicates that each CFS increase was associated with an increase in mortality in a linear fashion (25, 28). Our results, instead, are in contrast with this scenario.

This circumstance can be explained by the particular epidemiological and organizational context in which our research is based. The city of Parma was among the first European areas hit by a significant pandemic wave, in the first weeks of March 2020. The local healthcare system was put under extreme pressure, with significant number of patients seeking outpatient and inpatient care for fever and acute respiratory symptoms in a limited time frame (29, 30). Despite hospital care was promptly re-organized in order to face the pandemic emergency, with the institution of a medical hub, with variable number of beds dedicated to the care of patients with COVID-19 (14), the extreme demand of care among the population and the overload of medical community services could have determined the centralization to hospital only of those patients with extremely severe forms of respiratory failure. The emergence of the pandemic peak at the end of the winter season could have also favored SARS-CoV-2 transmission with very high viral loads, in comparison with geographical areas where the first pandemic peak arrived in spring, with hotter temperature and a climate less favorable to viral transmission (31, 32). If these assumptions are correct, then it seems reasonable that the severity of COVID-19 manifestations prevailed over pre-existing prognostic factors, like frailty, in influencing the risk of death. Some peculiarities of the COVID-19 presentation in older patients should be also considered. Age-related frailty is associated with decreased odds of presenting dyspnea, cough and fever at the onset of COVID-19 (33, 34). Atypical manifestations, like sudden functional decline, acute mental change, delirium, hypotension and dehydration, are instead more common. These characteristics, that were unfortunately unknown at the emergence of the first pandemic wave, could have determined reduced priority in access to hospital care for older frail patients. In any case, some multicenter studies have also underlined that frailty had only little incremental value in defining the prognosis of older patients hospitalized with COVID-19 during the earliest pandemic phases, in comparison with disease-related parameters (33, 35, 36). Frailty, instead, was a significant and strong predictor of post-COVID-19 functional impairment and post-discharge mortality in geriatric patients (37, 38). Interestingly, in a large multicenter study conducted in the Netherlands, atypical presentation of COVID-19 in older people was strongly associated with frailty, but not with increased risk of hospital mortality (39). The COVID-19 pandemic waves after the first were characterized by reduced severity of respiratory symptoms and reduced mortality (40–42). For the second and third wave, occurring in Italy before the widespread vaccination campaigns, the reason of this phenomenon, occurring despite the emergence of more aggressive viral strains, depends on earlier diagnosis, improvements in treatments, and better organization of care (40–42). From mid-2021 onwards, the progressive attenuation of COVID-19 severity and decrease of related mortality could be explained by the effects of vaccines (43, 44). The response to anti-SARS-CoV-2 vaccines and the duration of immunity is generally reduced in older people with frailty in comparison with adults, depending on specific comorbidity and polypharmacy profiles (45–47).

However, even with these limitations, COVID-19 vaccine administration is associated with a significant reduction of COVID-19 severity and mortality in geriatric patients (48). In this context, the association between frailty, measured by CFS, and mortality persisted (49–51). In particular, in a study conducted in 2362 patients over 70 years old, high CFS scores were associated with increased mortality across different pandemic waves, yet in a context of progressive mortality reduction after the introduction of vaccination campaigns (51).

It is noteworthy that the clinical complexity, in terms of comorbidities and prevalence of severe forms of frailty, was increased in vaccinated patients from the fourth pandemic wave, in comparison with patients admitted during the first wave. Despite protection granted by vaccines, older frail subjects from the fourth wave were probably more susceptible to symptomatic forms of COVID-19 requiring hospitalization than subjects without frailty (44). Data from our study reflect routine, unmonitored medical practice involving a broad spectrum of older patients with confirmed SARS-COV2 infection admitted to a single Internal Medicine hub during different phases of the pandemic. It can, therefore, provide insights into the natural history of SARS-COV2 and to be hypothesis generating. However, our investigation has several limitations that need to be addressed. First, the retrospective design does not allow to exclude selection bias. Residual confounding may remain, as certain potential confounding variables may have not been available or may have not had the desired level of granularity. The particular circumstances in which patients were hospitalized during the first pandemic peak, with unprecedented overload of the whole healthcare system, could have influenced the generalizability of our results. Finally, vaccinated patients from the fourth wave had a generally high burden of multimorbidity and clinical complexity not related to COVID-19, but to exacerbation of chronic diseases prompted by even mild SARS-CoV-2 infection (42). In this context, mortality of patients from the fourth wave cannot be certainly attributed to COVID-19, which is an important element that must be considered for a balanced interpretation of our results. Further research, including experimental and clinical studies, is needed to elucidate the underlying biological pathways and confirm causality.

## Conclusion

Our data suggest that, even in an older population with a high burden of frailty, the CFS may be unable to stratify the risk of hospital mortality for COVID-19 during an intense pandemic wave with significant workload for the care system. Conversely, the improvement in hospital care in the following waves and the effects of widespread vaccination campaigns restored the well-known association between CFS and mortality risk. These aspects should be considered when addressing preparedness of healthcare systems for future outbreaks.

## Data Availability

The raw data supporting the conclusions of this article will be made available by the authors, without undue reservation.

## References

[1] Guan WJ Ni ZY Hu Y Liang WH Ou CQ He JX. Clinical characteristics of coronavirus disease 2019 in China. N Engl J Med. (2020) 382:1708–20. doi: 10.1056/NEJMoa2002032, PMID: 32109013 PMC7092819

[2] Richardson S Hirsch JS Narasimhan M Crawford JM McGinn T Davidson KW. Presenting characteristics, comorbidities, and outcomes among 5700 patients hospitalized with COVID-19 in the new York City area. JAMA. (2020) 323:2052–9. doi: 10.1001/jama.2020.6775, PMID: 32320003 PMC7177629

[3] Chen T Wu D Chen H Yan W Yang D Chen G. Clinical characteristics of 113 deceased patients with coronavirus disease 2019: retrospective study. BMJ. (2020) 368:m1091. doi: 10.1136/bmj.m1091, PMID: 32217556 PMC7190011

[4] NICE. (2020). NICE updates rapid COVID-19 guideline on critical care. Available online at: https://www.nice.org.uk/news/article/nice-updates-rapid-covid-19-guideline-on-critical-care

[5] Rockwood K Song X MacKnight C Bergman H Hogan DB McDowell I. A global clinical measure of fitness and frailty in elderly people. CMAJ. (2005) 173:489–95. doi: 10.1503/cmaj.050051, PMID: 16129869 PMC1188185

[6] Morley JE Vellas B van Kan GA Anker SD Bauer JM Bernabei R. Frailty consensus: a call to action. J Am Med Dir Assoc. (2013) 14:392–7. doi: 10.1016/j.jamda.2013.03.022, PMID: 23764209 PMC4084863

[7] Hanlon P Nicholl BI Jani BD Lee D McQueenie R Mair FS. Frailty and pre-frailty in middle-aged and older adults and its association with multimorbidity and mortality: a prospective analysis of 493 737 UK biobank participants. Lancet Public Health. (2018) 3:e323–32. doi: 10.1016/S2468-2667(18)30091-4, PMID: 29908859 PMC6028743

[8] Romero-Ortuno R Wallis S Biram R Keevil V. Clinical frailty adds to acute illness severity in predicting mortality in hospitalized older adults: an observational study. Eur J Intern Med. (2016) 35:24–34. doi: 10.1016/j.ejim.2016.08.033, PMID: 27596721

[9] Smart R Carter B McGovern J Luckman S Connelly A Hewitt J. Frailty exists in younger adults admitted as surgical emergency leading to adverse outcomes. J Frailty Aging. (2017) 6:219–23. doi: 10.14283/jfa.2017.28, PMID: 29165541

[10] Gilbert T Neuburger J Kraindler J Keeble E Smith P Ariti C. Development and validation of a hospital frailty risk score focusing on older people in acute care settings using electronic hospital records: an observational study. Lancet. (2018) 391:1775–82. doi: 10.1016/S0140-6736(18)30668-8, PMID: 29706364 PMC5946808

[11] Yang Y Luo K Jiang Y Yu Q Huang X Wang J. The impact of frailty on COVID-19 outcomes: a systematic review and Meta-analysis of 16 cohort studies. J Nutr Health Aging. (2021) 25:702–9. doi: 10.1007/s12603-021-1611-9, PMID: 33949641 PMC7933604

[12] Meschi T Rossi S Volpi A Ferrari C Sverzellati N Brianti E. Reorganization of a large academic hospital to face COVID-19 outbreak: the model of Parma, Emilia-Romagna region, Italy. Eur J Clin Investig. (2020) 50:e13250. doi: 10.1111/eci.13250, PMID: 32367527 PMC7262013

[13] Siniscalchi C Ticinesi A Nouvenne A Guerra A Parise A Finardi L. Non-invasive ventilation support during hospitalization for SARS-CoV-2 and the risk of venous thromboembolism. J Clin Med. (2024) 13:2737. doi: 10.3390/jcm13102737, PMID: 38792278 PMC11122199

[14] Daughety MM Morgan A Frost E Kao C Hwang J Tobin R. COVID-19 associated coagulopathy: thrombosis, hemorrhage and mortality rates with an escalated-dose thromboprophylaxis strategy. Thromb Res. (2020) 196:483–5. doi: 10.1016/j.thromres.2020.10.004, PMID: 33091700 PMC7557260

[15] Falk Erhag H Guðnadóttir G Alfredsson J Cederholm T Ekerstad N Religa D. The association between the Clinical Frailty Scale and adverse health outcomes in older adults in acute clinical settings - a systematic review of the literature. Clin Interv Aging. (2023) 18:249–61. doi: 10.2147/CIA.S388160, PMID: 36843633 PMC9946013

[16] Granata N Vigoré M Steccanella A Ranucci L Sarzi Braga S Baiardi P. The Clinical Frailty Scale (CFS) employment in the frailty assessment of patients suffering from non-communicable diseases (NCDs): a systematic review. Front Med. (2022) 9:967952. doi: 10.3389/fmed.2022.967952, PMID: 36052327 PMC9425100

[17] Hewitt J Carter B Vilches-Moraga A Quinn TJ Braude P Verduri A. The effect of frailty on survival in patients with COVID- 19 (COPE): a multicentre, European, observational cohort study. Lancet Public Health. (2020) 5:e444–51. doi: 10.1016/S2468-2667(20)30146-8, PMID: 32619408 PMC7326416

[18] De Smet R Mellaerts B Vandewinckele H Lybeert P Frans E Ombelet S. Frailty and mortality in hospitalized older adults with COVID-19: retrospective observational study. J Am Med Dir Assoc. (2020) 21:928–932.e1. doi: 10.1016/j.jamda.2020.06.008, PMID: 32674821 PMC7280137

[19] Hägg S Jylhäva J Wang Y Xu H Metzner C Annetorp M. Age, frailty, and comorbidity as prognostic factors for short-term outcomes in patients with coronavirus disease 2019 in geriatric care. J Am Med Dir Assoc. (2020) 21:1555–1559.e2. doi: 10.1016/j.jamda.2020.08.014, PMID: 32978065 PMC7427570

[20] Marengoni A Zucchelli A Vetrano DL Armellini A Botteri E Nicosia F. Beyond chronological age: frailty and multimorbidity predict in-hospital mortality in patients with coronavirus disease 2019. J Gerontol A Biol Sci Med Sci. (2021) 76:e38–45. doi: 10.1093/gerona/glaa291, PMID: 33216846 PMC7717138

[21] Sablerolles RSG Lafeber M van Kempen JAL van de Loo BPA Boersma E Rietdijk WJR. Association between Clinical Frailty Scale score and hospital mortality in adult patients with COVID-19 (COMET): an international, multicentre, retrospective, observational cohort study. Lancet Healthy Longev. (2021) 2:e163–70. doi: 10.1016/S2666-7568(21)00006-4, PMID: 33655235 PMC7906710

[22] Romero Aliberti MJ Szlejf C Avelino-Silva VI Suemoto CK Apolinario D Dias MB. COVID-19 is not over and age is not enough: using frailty for prognostication in hospitalized patients. J Am Geriatr Soc. (2021) 69:1116–27. doi: 10.1111/jgs.17146, PMID: 33818759 PMC8251205

[23] Bavaro DF Diella L Fabrizio C Sulpasso R Bottalico IF Calamo A. Peculiar clinical presentation of COVID-19 and predictors of mortality in the elderly: a multicentre retrospective cohort study. Int J Infect Dis. (2021) 105:709–15. doi: 10.1016/j.ijid.2021.03.021, PMID: 33722685 PMC7967397

[24] Blomaard LC van der Linden CMJ van der Bol J Jansen SWM Polinder-Bos HA Willems HC. Frailty is associated with in- hospital mortality in older hospitalized COVID-19 patients in the Netherlands: the COVID-OLD study. Age Ageing. (2021) 50:631–40. doi: 10.1093/ageing/afab018, PMID: 33951156 PMC7929372

[25] Kastora S Kounidas G Perrott S Carter B Hewitt J Myint PK. Clinical Frailty Scale as a point of care prognostic indicator of mortality in COVID-19: a systematic review and meta- analysis. EClinicalMedicine. (2021) 36:100896. doi: 10.1016/j.eclinm.2021.100896, PMID: 34036252 PMC8141355

[26] Cecchini S Di Rosa M Soraci L Fumagalli A Misuraca C Colombo D. Chest X-ray score and frailty as predictors of in-hospital mortality in older adults with COVID-19. J Clin Med. (2021) 10:2965. doi: 10.3390/jcm10132965, PMID: 34279449 PMC8268684

[27] Rebora P Focà E Salvatori A Zucchelli A Ceravolo I Ornago AM. The effect of frailty on in-hospital and medium- term mortality of patients with COronaVirus Disease-19: the FRACOVID study. Panminerva Med. (2022) 64:24–30. doi: 10.23736/S0031-0808.21.04506-7, PMID: 34761887

[28] Pranata R Henrina J Lim MA Lawrensia S Yonas E Vania R. Clinical Frailty Scale and mortality in COVID-19: a systematic review and dose-response meta-analysis. Arch Gerontol Geriatr. (2021) 93:104324. doi: 10.1016/j.archger.2020.104324, PMID: 33352430 PMC7832565

[29] Caminiti C Maglietta G Meschi T Ticinesi A Silva M Sverzellati N. Effects of the COVID-19 epidemic on hospital admissions for non-communicable diseases in a large Italian university- hospital: a descriptive case-series study. J Clin Med. (2021) 10:880. doi: 10.3390/jcm10040880, PMID: 33669906 PMC7924591

[30] Ticinesi A Nouvenne A Cerundolo N Parise A Prati B Guerra A. Trends of COVID-19 admissions in an Italian hub during the pandemic peak: large retrospective study focused on older subjects. J Clin Med. (2021) 10:1115. doi: 10.3390/jcm10051115, PMID: 33800020 PMC7962097

[31] Liu X Huang J Li C Zhao Y Wang D Huang Z. The role of seasonality in the spread of COVID-19 pandemic. Environ Res. (2021) 195:110874. doi: 10.1016/j.envres.2021.110874, PMID: 33610582 PMC7892320

[32] Townsend JP Hassler HB Lamb AD Sah P Alvarez Nishio A Nguyen C. Seasonality of endemic COVID-19. MBio. (2023) 14:e0142623. doi: 10.1128/mbio.01426-23, PMID: 37937979 PMC10746271

[33] Eiras Poco PC Romero Alberti MJ Bacchini Dias M de Fatima Takahashi S Leonel FC Altona M. Divergent: age, frailty, and atypical presentations of COVID-19 in hospitalized patients. J Gerontol A Biol Sci Med Sci. (2021) 76:e46–51. doi: 10.1093/gerona/glaa280, PMID: 33151305 PMC7665317

[34] Trevisan C Remelli F Fumagalli S Mossello E Okoye C Bellelli G. COVID-19 as a paradigmatic model of the heterogeneous disease presentation in older people: data from the GeroCovid observational study. Rejuvenation Res. (2022) 25:129–40. doi: 10.1089/rej.2021.0063, PMID: 35570723

[35] Lozano-Montoya I Quezada-Feijoo M Jaramillo-Hidalgo J Garmendia-Prieto B Lisette-Carrillo P Gómez-Pavón FJ. Mortality risk factors in a Spanish cohort of oldest-old patients hospitalized with COVID-19 in an acute geriatric unit: the OCTA-COVID study. Eur Geriatr Med. (2021) 12:1169–80. doi: 10.1007/s41999-021-00541-0, PMID: 34287813 PMC8294271

[36] Owen RK Conroy SP Taub N Jones W Bryden D Pareek M. Comparing associations between frailty and mortality in hospitalised older adults with or without COVID-19 infection: a retrospective observational study using electronic health records. Age Ageing. (2021) 50:307–16. doi: 10.1093/ageing/afaa167, PMID: 32678866 PMC7454252

[37] Geriatric Medicine Research Collaborative, Covid Collaborative Welch C. Age and frailty are independently associated with increased COVID-19 mortality and increased care needs in survivors: results of an international multi-Centre study. Age Ageing. (2021) 50:617–30. doi: 10.1093/ageing/afab026, PMID: 33543243 PMC7929433

[38] Vlachogiannis NI Baker KF Georgiopoulos G Lazaridis C Schim van der Loeff I Hanrath AT. Clinical frailty, and not features of acute infection, is associated with late mortality in COVID-19: a retrospective cohort study. J Cachexia Sarcopenia Muscle. (2022) 13:1502–13. doi: 10.1002/jcsm.12966, PMID: 35257497 PMC9088314

[39] Van Son JE Kahn KCP van der Bol JM Barten DG Blomaard LC van Dam C. Atypical presentation of COVID-19 in older patients is associated with frailty but not with adverse outcomes. Eur Geriatr Med. (2023) 14:333–43. doi: 10.1007/s41999-022-00736-z, PMID: 36749454 PMC9902812

[40] Smits RAL Trompet S van der Linden CMJ van der Bol JM Jansen SWM Polinder-Bos HA. Characteristics and outcomes of older patients hospitalised for COVID-19 in the first and second wave of the pandemic in the Netherlands: the COVID-OLD study. Age Ageing. (2022) 51:afac048. doi: 10.1093/ageing/afac048, PMID: 35235650 PMC8890695

[41] Verduri A Short R Carter B Braude P Vilches-Moraga A Quinn TJ. Comparison between first and second wave of COVID-19 outbreak in older people: the COPE multicentre European observational cohort study. Eur J Pub Health. (2022) 32:807–12. doi: 10.1093/eurpub/ckac108, PMID: 35997587 PMC9452163

[42] Ticinesi A Parise A Nouvenne A Cerundolo N Prati B Guerra A. Insights from comparison of the clinical presentation and outcomes of patients hospitalized with COVID-19 in an Italian internal medicine ward during first and third wave. Front Med. (2023) 10:1112728. doi: 10.3389/fmed.2023.1112728, PMID: 36817786 PMC9928966

[43] Smits RAL van Raaij BFM Trompet S van der Linden CMJ van der Bol JM Jansen SWM. Differences in characteristics and outcomes of older patients hospitalized for COVID-19 after introduction of vaccination. Eur Geriatr Med. (2024) 15:941–9. doi: 10.1007/s41999-024-01002-0, PMID: 38861241 PMC11377515

[44] Ticinesi A Parise A Cerundolo N Nouvenne A Prati B Chiussi G. Multimorbidity and frailty are the key characteristics of patients hospitalized with COVID-19 breakthrough infection during delat variant predominance in Italy: a retrospective study. J Clin Med. (2022) 11:5442. doi: 10.3390/jcm11185442 36143095 PMC9503996

[45] Tang F Hammel IS Andrew MK Ruiz JG. Frailty reduces vaccine effectiveness against SARS-CoV-2 infection: a test-negative case control study using national VA data. J Nutr Health Aging. (2023) 27:81–8. doi: 10.1007/s12603-023-1885-1, PMID: 36806862 PMC9893970

[46] Trevisan C Raparelli V Malara A Abbatecola AM Noale M Palmieri A. Sex differences in the efficacy and safety of SARS-CoV-2 vaccination in residents of long-term care facilities: insights from the GeroCovid vax study. Intern Emerg Med. (2023) 18:1337–47. doi: 10.1007/s11739-023-03283-y, PMID: 37120663 PMC10148701

[47] Trevisan C Haxhiaj L Malara A Abbatecola A Fedele G Palmieri A. Polypharmacy and antibody response to SARS-CoV-2 vaccination in residents of long-term care facilities: the GeroCovid vax study. Drugs Aging. (2023) 40:1133–41. doi: 10.1007/s40266-023-01075-9, PMID: 37938521

[48] Antonelli M Penfold RS Dos Santos CL Sudre C Rjoob K Murray B. SARS-CoV-2 infection following booster vaccination: illness and symptom profile in a prospective, observational community-based case-control study. J Infect. (2023) 87:506–15. doi: 10.1016/j.jinf.2023.08.009, PMID: 37777159 PMC7618294

[49] Andrew MK Godin J LeBlanc J Boivin G Valiquette L McGeer A. Older age and frailty are associated with higher mortality but lower ICU admission with COVID-19. Can Geriatr J. (2022) 25:183–96. doi: 10.5770/cgj.25.546, PMID: 35747412 PMC9156416

[50] Martí-Pastor A Moreno-Perez O Lobato-Martínez E Valero-Sempere F Amo-Lozano A Martínez-García MÁ. Association between Clinical Frailty Scale (CFS) and clinical presentation and outcomes in older inpatients with COVID-19. BMC Geriatr. (2023) 23:1. doi: 10.1186/s12877-022-03642-y, PMID: 36593448 PMC9806809

[51] Van Raaj BFM Noordam R Smits RAL van der Klei VMGTH Jansen SWM van der Linden CMJ. Preparing for future pandemics: frailty associates with mortality in hospitalized older people during the entire COVID-19 pandemic, a Dutch multicentre cohort study. Eur Geriatr Med. (2024) 15:951–9. doi: 10.1007/s41999-024-01001-1, PMID: 38849648 PMC11377458

